# Alterations in Cellular Processes Involving Vesicular Trafficking and Implications in Drug Delivery

**DOI:** 10.3390/biomimetics3030019

**Published:** 2018-07-24

**Authors:** Silvia Muro

**Affiliations:** 1 Institute for Bioscience and Biotechnology Research and Fischell Department of Bioengineering, University of Maryland, College Park, MD 20742, USA; 2 Catalan Institution for Research and Advanced Studies (ICREA), 08010 Barcelona, Spain; 3 Institute for Bioengineering of Catalonia (IBEC) of the Barcelona Institute of Science and Technology (BIST), 08028 Barcelona, Spain; smuro@ibecbarcelona.eu; Tel.: +34-934-020-440

**Keywords:** cellular vesicles, vesicle fusion, fission and intracellular trafficking, drug delivery systems and nanomedicines, transcytosis and endocytosis of drugs carriers, disease effects on vesicular trafficking, drug effects on vesicular trafficking, role of the biological environment

## Abstract

Endocytosis and vesicular trafficking are cellular processes that regulate numerous functions required to sustain life. From a translational perspective, they offer avenues to improve the access of therapeutic drugs across cellular barriers that separate body compartments and into diseased cells. However, the fact that many factors have the potential to alter these routes, impacting our ability to effectively exploit them, is often overlooked. Altered vesicular transport may arise from the molecular defects underlying the pathological syndrome which we aim to treat, the activity of the drugs being used, or side effects derived from the drug carriers employed. In addition, most cellular models currently available do not properly reflect key physiological parameters of the biological environment in the body, hindering translational progress. This article offers a critical overview of these topics, discussing current achievements, limitations and future perspectives on the use of vesicular transport for drug delivery applications.

## 1. Introduction

Formation, fusion, fission and trafficking of membranous vesicles are fundamental processes which commonly occur in cells within the body and regulate both basic and specialized functions required for life [1,2]. These include cellular uptake and mobilization of nutrients to enable their metabolic processing and that of foreign pathogens and substances for their degradation; trafficking of newly synthesized molecules and complexes to their various intracellular locations; secretion of toxic compounds, communication signals, extracellular vesicles, or specialized molecules; organelle formation, as well as intra- and trans-organelle communication; subcellular location of signaling platforms, as well as processing and termination of these cascades; modification and recycling of lipid and protein elements in the plasmalemma and intracellular membranes; autophagic and apoptotic processes and many other functions [1,2]. Among them, the best characterized vesicular processes are those that involve vesicular trafficking from the cell interior to the plasma membrane (the secretory route) and those that relate to vesicular trafficking from the plasma membrane to the cell interior (the endocytic route), both of which are interconnected [1,2].

These vesicular transport mechanisms are often investigated and exploited as portals to facilitate the delivery of therapeutic compounds either across cellular barriers that separate compartments in the body (e.g., via transcytosis) or into the cells which necessitate therapeutic intervention (via various endocytic pathways) [3,4,5]. Therefore, their relevance pertains to both the fundamental functioning and maintenance of cellular and body homeostasis and the practical realm of translational applications. In this translational context, it is important to emphasize that the cell receptors and/or pathways engaged may respond differently compared to their natural function. For instance, a non-endocytic marker may be prone to endocytosis when engaged by a drug conjugate or carriers or, vice versa, the delivery system may decrease uptake of an otherwise readily endocytic receptor [6,7,8,9]. Furthermore, the size, shape, stiffness, deformability, surface properties and targeting valency of drug conjugates and carriers additionally modulate endocytic activity, affecting uptake efficiency and final intracellular destination, sometimes in unpredictable manners [10,11,12,13,14,15,16,17,18].

However, how vesicular transport is regulated in disease or whether transport alterations arise as side effects of drug delivery systems are questions far less studied. Because the mechanisms and cellular machinery that regulate uptake and trafficking via vesicular routes are highly complex and tightly regulated, vesicular transport processes are rather susceptible to suffering alterations [2,4]. Under this context, the study of potential changes in these routes is significant in order to understand their pathological implications and contribution to disease outcomes, as well as to select the most amenable routes available for drug delivery in particular disease settings. In addition, hijacking these pathways may render detrimental consequences to the natural vesicular transport of endogenous molecules in the body.

This article aims to review this topic, offering both fundamental information about the regulation of said vesicular transport pathways [2] and their translational application as routes to access across cellular barriers that separate body compartments and to enter cells in the context of drug delivery [3,4,5]. Most particularly, the focus is on raising awareness on the alterations that said pathways often suffer under various conditions relevant to their translational use. This encompasses the effects of the pathological syndrome itself, the use of particular drugs and supplements, and side effects exerted by the drug carriers employed, all of which alter the availability of cellular transport routes for drug delivery applications [19,20,21,22]. How different cellular models used in research impact the efficacy of said pathways and, hence, the observed delivery outcome is also discussed [23], along with considerations on the selection and need of systems which more closely reproduce key physiological parameters of the biological environment [24]. The achievements, limitations and future perspectives on these topics are discussed under a critical perspective.

## 2. Vesicular Transport in Cells

### 2.1. Uptake via Pathways Which Employ Vesicular Trafficking

**Figure 1:**
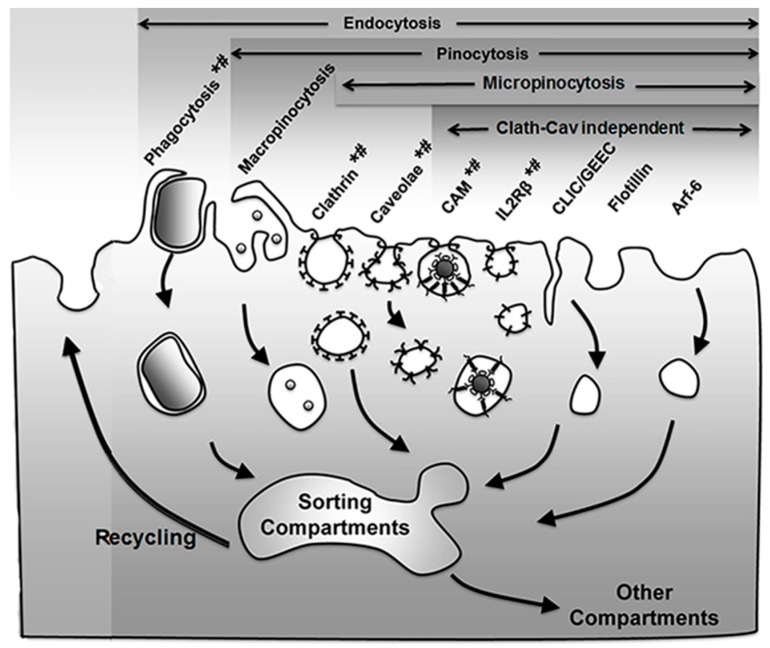
Endocytic pathways. Endocytosis via membranous vesicles encompasses several mechanisms where the plasmalemma internalizes objects (phagocytosis) and extracellular fluid (pinocytosis) in intracellular vesicles. Some vesicles concentrate receptors that mediate uptake of specific ligands via receptor-mediated endocytosis (marked by *), while others (e.g., macropinocytosis) largely internalize fluid and solutes by non-specific means. Pinocytosis is subdivided in macro- and micropinocytosis depending on the size of the vesicles that form and the latter can be mediated by classical (clathrin- and caveolae-mediated pathways) or non-classical (clathrin and caveolae independent) routes, all of which can be classified as per their dynamin dependence (marked by ^#^). Apart from the trafficking destinations shown, some markers (e.g., platelet–endothelial cell adhesion molecule 1 (PECAM-1) and intercellular adhesion molecule 1 (ICAM-1) associated with the cell adhesion molecule (CAM)-mediated pathway) may shuttle back and forth between the cell surface and a subplasmalemma vesicular compartment whose membrane is continuous with the plasmalemma. Adapted and reproduced with permission from Figure 12.2 in [26]. Copyright 2016 Pan Stanford.

As mentioned in the Introduction, the group of processes resulting in the formation of vesicles budding off the plasma membrane into the cytosol, which may engulf extracellular material and/or simply carry membrane components for recycling, is generically termed endocytosis [25]. Several mechanisms regulate this form of transport, which can be used to access cells for drug delivery applications [3,4,5] (Figure 1 and Table 1).

An initial classification of these pathways divided them into phagocytosis and pinocytosis, referring to the nature of the materials being taken up as either bulky objects or fluid phase, respectively. Phagocytosis is mostly utilized by cells of the immune system to remove pathogens and particulate matter, although endothelial and other cells can also exert this action [27,28]. This is mediated by receptors on their plasmalemma which recognize certain modifications or common domains in groups of molecules and molecular patterns, including adhesion molecules, lipids, carbohydrates, antibodies, which are respectively recognized by integrins, scavenger receptors, mannose receptors, Fc receptors, etc. [28]. Binding of said ligands to their receptors recruits cytoskeletal elements, most precisely actin cups, which help with the membrane engulfment of the bound cargo [28]. The vesicles that form through this process, called phagosomes, are most often targeted to lysosomal-like compartments in the cell, where the cargo is degraded [28]. Applications of this pathway in the realm of drug delivery include, for instance, the use of liposomes to deliver antibiotics to macrophages, tested in mouse models, to treat bacterial and parasitic infections such as those caused by *Salmonella typhimurium*, *Mycobacterium tuberculosis*, *Listeria monocytogenes* [29]. Another example is that of polymeric nanoparticles aimed to deliver cytotoxic agents in the context of hepatic tumors. This is the case for poly(alkylcyanoacrylate) (PACA) nanoparticles loaded with doxorubicin (Onxeo’s Livatag^®^) [30]. Livatag^®^ was granted orphan drug status both in the U.S. and Europe and entered phase III clinical trials in 2013 for treatment of hepatocellular carcinoma. Unfortunately, the formulation failed to meet the primary end point, which aimed to show improved survival vs. patients treated with classical drugs.

As described above, pinocytosis refers to the vesicular uptake of fluid into a cell, which may occur mediated by different pathways [25]. A generic classification distinguishes between pinocytic mechanisms where large micrometer-range vs. small submicrometer vesicles form (macro- vs. micropinocytosis, respectively) [25]. In the first case, uptake does not require mediation by membrane receptors, while the second most often requires induction by particular ligands binding to their membrane receptors [25]. As a note, not only membrane-bound ligands can enter endocytic vesicles via these mechanisms but also small solutes which may be concentrated in the extracellular milieu may be passively incorporated as fluid [25].

The vesicles that form via macropinocytosis are called macropinosomes and are also characteristic of immune cells, such as antigen-presenting ones, although this can be induced in additional cell types [27]. Because of the large deformations, ruffles and other structures required to form at the plasmalemma in this pathway, macropinocytosis is highly dependent on the actin cytoskeleton. Macropinosmes can follow several trafficking routes, most commonly to lysosomes for degradation but also to recycling pathways [31]. Noteworthy, vesicles which form via this route tend to be leaky, which can be an advantage for drug delivery, as long and the target cells possess the ability to internalize materials through this pathway [31]. Examples of drug delivery approaches exploiting this mechanism include strategies which used cell-penetrating peptides for gene delivery and treatment of leukemia [32].

With regard to micropinocytic processes, these can be regulated via multiple pathways, including clathrin- or caveolae-mediated, as well as clathrin- and caveolae-independent ones. The most studied example is clathrin-mediated endocytosis, a mechanism rather ubiquitous of most cells in the body [25]. It is characterized by formation of ≈100–150 nm in diameter vesicles coated with a scaffold protein called clathrin [25]. Many receptors and other molecules present on the plasma membrane are internalized by this mechanism, such as the receptors for insulin, low-density lipoprotein (LDL), transferrin, several growth factors and some adhesion molecules [25,33]. Materials which enter cells via this pathway may traffic to lysosomal compartments, recycling routes, or transcytosis across the cell body in the case of polarized cells which separate body compartments, such as for epithelial or endothelial monolayers [33,34]. Examples of drug delivery systems using this route include that of PACA nanoparticles coated with transferrin, which were used for delivery of paclitaxel to S-180 tumor-bearing mice [29], or that of similarly targeted albumin nanoparticles loaded with azidothymidine, an antiretroviral agent, which was delivered to the brain in rat models of human immunodeficiency virus (HIV)/acquired immune deficiency syndrome (AIDS) [35].

Another endocytic pathway abundant in many, but not all, cell types is the one mediated by caveoli [27]. Caveolae are constitutive flask-shaped invaginations of the plasma membrane, which contain a “pseudo-coat” formed by a transmembrane protein called caveolin [36]. These invaginations concentrate at lipid-dense microdomains and are ≈60–80 nm in diameter [36]. Materials internalized via this route can traffic to lysosomes, the Golgi and rough endoplasmic reticulum (ER) but most commonly result in transcytosis across polarized cells [37]. Uptake of ligands targeting ganglioside GM1, aminopeptidases N and P, the albumin-binding receptor gp60, plasmalemma vesicle associated protein (PLVAP) and others occurs by this route [4,10,27]. Another example, although the mechanism is still under debate, is that of folate, broadly tested for drug delivery applications involving drug conjugates, liposomes, polymer nanoparticles, etc. [8]. The strategy of targeting caveolar pathways has been shown to enhance the accumulation of therapeutic and diagnostic agents in tumor and inflammatory settings [8,10,38].

Less known is a group of routes commonly termed clathin- and caveolae-independent pathways, which may be dynamin-dependent or -independent (note: clathrin and caveolae-dependent routes and some examples of macropinocytosis, also depend on dynamin; see Section 2.2 for a discussion on the role of this molecule). Dynamin-dependent examples of these non-classical pathways are that of the uptake of interleukin (IL) receptors (i.e., IL2Rβ [39], IL4Rα [40], IL15Rα [41]), some flotillin-associated receptors [42], or the cell adhesion molecule (CAM)-mediated pathway by which intercellular adhesion molecule 1 (ICAM-1) or platelet-endothelial cell adhesion molecule 1 (PECAM-1) are internalized, which is exploited by some viral pathogens for cell invasion [6,43]. Pathways which seem to operate in the absence of dynamin are that of the clathrin-independent carrier/glycosylphosphatidylinositol-anchored protein (GPI-AP)-enriched early endosomal compartment (CLIC/GEEC) [44], the adenosine-diphosphate (ADP) ribosylation factor-6 (Arf6) pathway and the flotillin-1 dependent pathway [45]. While these routes are still under investigation and their biologically regulation is less understood, both IL2-targeting and, most profusely, the CAM pathway are being explored for drug delivery in the context of therapy-refractory cutaneous T-cell lymphoma (IL2-mediated) as well as antioxidative, anti-inflammatory approaches [43,45].

### 2.2. Regulation of Intracellular Vesicular Trafficking

**Figure 2:**
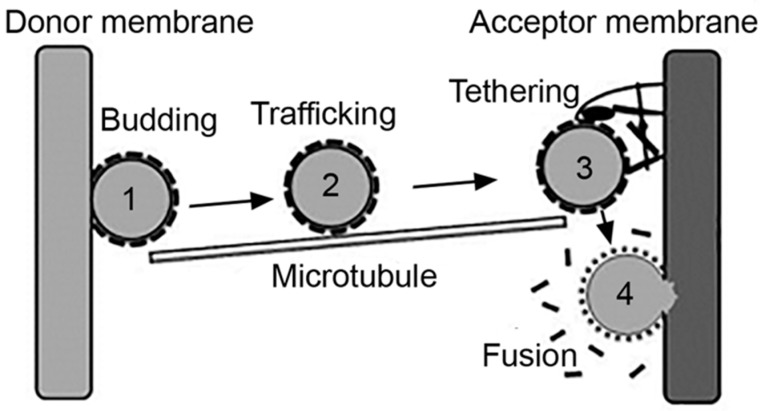
Vesicular transport. Intracellular vesicles transport cargo between the plasmalemma and organelles or from an organelle to another, which encompasses the processes of: (**1**) budding of a nascent vesicle from the donor membrane and pinching off into the cytosol; (**2**) trafficking aided by cytoskeletal elements; (**3**) tethering of the vesicle to the acceptor membrane; and (**4**) fusion to deliver cargo to the acceptor compartment. Adapted and reproduced with permission from Figure 3.2 in [2]. Copyright 2016 Pan Stanford.

Intracellular vesicles act as natural carriers which transport cargo between the plasmalemma and an “organelle,” as the case of the endosomal network and also between two cellular organelles, such as between the ER and the Golgi network [46]. Said cargo may: (i) be contained in the lumen of the vesicle, such as antigens and drugs internalized via pinocytosis; (ii) be a part of the membranous counterpart of the vesicle, for example, lipids and integral proteins such as receptors being recycled, or drugs which partition within the membrane; or (iii) be transported via interacting with a membrane component, such as a ligand or targeted drug-carrier bound to their receptors [2,4]. In all cases, effective transport of cargo implies the formation of a vesicle and fission from a donor membrane, the transport and targeting of the vesicle toward an acceptor membrane and the final tethering and fusion of the vesicle to said acceptor membrane, for release of the transported cargo [46] (Figure 2).

In most examples but not all, the first steps leading to vesicle formation require an inducing signal, such as a ligand binding to its membrane receptor [3,4,5]. This involves conformational changes of said receptor, so that its cytoplasmic domain exposes sequences which recruit additional signaling molecules and, in many cases, protein adaptors and coats [1]. Assembly of these protein complexes help modulate the membrane curvature, which initiates the formation of a nascent bud [47]. This structure progresses as more adaptors and coat proteins assemble at the site, which simultaneously helps concentrate receptors and bound ligands [46]. Finally, fission of the nascent vesicle from the donor membrane must occur, which is commonly mediated by dynamin [48]. This is a molecular motor with GTPase activity, which polymerizes into oligomeric structures that form a helical tube around the vesicle neck. Guanosine triphosphate (GTP) to guanosine diphosphate (GDP) hydrolysis generates conformational changes leading to the twisting and tightening of the dynamin helix, which results in the vesicle pinching off the donor membrane into the cytoplasm [49].

Subsequently, the vesicle is transported from the donor site toward the acceptor membrane. This is mediated by cytoskeletal elements, most often microtubules [50]. Molecular motors bind and bridge between the vesicle and the microtubule and provide the dynamic forces which result in the physical movement of the vesicle across the cytoplasmic space that separates donor and acceptor sites [50]. Vesicles which must be transported from more peripheral to more central locations in the cell use dynein motors, which move toward the minus end of microtubules [50]. Instead, those that must be transported from more central regions to the cell periphery use kinesin motors, which move toward the plus end of these cytoskeletal elements [50]. In some cases, myosin motors and actin filaments can provide for this function [51]. Where distances are less compelling, as (speculatively) the case of transcytosis across very thin endothelial cells or uptake and recycling through the subplasmalemmal endosomal network, vesicular transport is also possible without the involvement of cytoskeletal elements [2].

Once at the immediate vicinity of the acceptor site, tethering factors assemble into multimeric complexes, which help anchor the vesicle to the membrane [52]. These complexes provide both structural support as well interaction with regulatory proteins which help in docking and fusion. Said assembly and regulation is largely mediated by monomeric G proteins called Rabs. Specific members of this family are anchored to specific membranes via lipidic prenyl groups. They can also interact with cytoskeletal elements and motors regulating transport of cytoskeletal tracks [52]. Rabs also exert GTPase activity and their action is regulated depending on their GTP- vs. GDP-bound states [52,53]. Guanine nucleotide-exchange factors (GEFs) and GTPase-activating proteins (GAPs) are additional factors which exchange both types of nucleotides and regulate the GTPase activity of Rabs, respectively [52]. Many accessory proteins are additionally involved with specific Rabs and their downstream effectors. These proteins may, for instance, exchange the type of Rab present on a membranous compartment to modulate its maturation into a downstream compartment, such as the case of sequential recruitment of Rab5 followed by Rab7, which associates to maturation of early endosomes into late endosomes [54].

At this stage, fusion between the membrane of the transported vesicle and the acceptor site is mediated by other protein complexes termed soluble *N*-ethylmaleimide-sensitive factor (NSF) attachment protein (SNAP) receptors (SNAREs) [52,55]. SNARE complexes are located on all cellular membranes (the plasmalemma and that of organelles), although each location is marked by particular SNAREs, which generates the specificity of vesicular targeting to selected locations [56]. The SNAREs located on a vesicle specifically bind to cognate SNAREs on the acceptor membrane, which triggers complex conformational changes that provide the necessary energy to keep the vesicular and acceptor membranes in intimate contact, despite natural repulsive forces which tend to keep membranes apart [57]. It has been mathematically estimated that interaction of two to three SNARE pairs is sufficient to provide membrane fusion [42]. After fusion between the vesicular and acceptor membranes, SNARE complexes are actively disengaged by NSF and SNAP proteins, which free SNAREs to enable them to engage in future fusion events [58,59].

### 2.3. Intracellular Trafficking Subsequent to Vesicular Uptake

After uptake occurs and through the molecular machinery described in Section 2.2, most vesicles internalized within the cell (and, hence, their cargo) are mobilized through sorting compartment to different destinations. Most commonly this involves trafficking to early endosomes, recycling endosomes, late endosomes and lysosomes [60]. Early endosomes constitute the first trafficking stage after uptake for most routes, although it is more abundantly associated with the clathrin-mediated pathway. This compartment has pH of ≈6.5, which favors ligand-receptor dissociation [60]. Many receptors enter recycling endosomes and traffic back to the plasmalemma, where they can remain “latent” or engage into a new round of binding and uptake if more ligand molecules remain available [60]. Most cargo and in some instances receptors, enter late endosomes and, subsequently lysosomes. Both compartments are acidic and contain degradative enzymes but the concentration of certain components such as lysosome-associated membrane proteins (LAMPs), lack of others such as mannose-6-phosphate receptors and a greater density are characteristics more common to lysosomes [61]. In these compartments, the pH lowers to ≈5.5 and ≈4.5, respectively, which enables the catalytic activity of said degradative enzymes [61]. Compartments derived from uptake mechanisms, such as phagosomes, macropinosomes, and “caveosomes”, as well as intracellular compartments associated with other processes, such as autophagosomes and trans-Golgi vesicles, can also interact with and deliver cargo to late endosomes and lysosomes [4]. Additionally, in the case of PECAM-1 and ICAM-1 expressed on endothelial cells, it has been described that these markers can recycle back and forth a subplasmalemma compartment of interconnected invaginations, which appears to be in continuity with the plasma membrane [62,63]. Uptake of affinity moieties to these markers, such as antibody fragments is followed by trafficking through this route, avoiding late endosomes and lysosomal compartments, which represent the final destination for most multimeric conjugates or nanoparticles [62,63,64].

In the context of drug delivery, lysosomal transport may be an advantage in some instances. For example, in the case of membrane-permeable drugs with strong side effects, such as doxorubicin, conjugation or encapsulation diminishes their toxicity and switch their access to cells from passive diffusion to active endocytosis. As such, the use of pH- and/or enzyme-responsive linkers or carriers enable drug release in this compartment, resulting in diffusion throughout the target while minimizing uncontrolled diffusion prior reaching the target [65,66,67]. Controlled drug release can be also achieved where lysosomal pH is used to induce porosity or volumetric changes in the drug carrier [68,69]. In other cases, such as for treatment of lysosomal disorders, this route represents an ideal avenue to access the therapeutic target [70].

Conversely, most often, entry in the endolysosomal route renders drugs entrapped in these vesicular compartments and, depending on the drug lability to pH and enzymatic degradation, inactive [70]. Because of this, many strategies have been designed to exploit lysosomal properties to overcome this obstacle. This is the case for lytic peptides derived from bacteria, for example, *L. monocytogenes*’ listeriolysin O which forms a membrane pore at acidic pH, through which small drugs could escape into the cytosol [71]. Natural or designed fusogenic peptides, such as those derived from West Nile virus (WNV), HIV, influenza, etc., or synthetic GALA or KALA peptides, have also been investigated for their ability to fuse and destabilize the membrane of these terminal compartments [72,73]. In addition to these means, many polycationic systems have been designed which buffer the endosomal pH and result in osmotic swelling in endosomes (e.g., by enhancing Cl^−^ influx [74]). Some liposomal formulations are also designed to be pH sensitive, so that they are stable at neutral pH but acquire fusogenic properties at the acidic pH of endolysosomal compartments, leading to cytosolic delivery [75,76]. Several types of deoxyribonucleic acid (DNA)-built carriers also seem to escape endosomes, although the mechanism for this activity is not well understood [77,78,79].

Although much less commonly, apart from this sorting through the endolysosomal system, both clathrin- and caveolae-mediated endocytosis processes have been associated with trafficking to other subcellular destinations, such as the Golgi or the ER [80]. This is the case for various pathogen toxins, for example, cholera toxin or shiga toxin, which appear to be able to follow this path, upon which some drug delivery strategies have been developed [81,82].

Finally, in the case of cellular monolayers that separate body compartments, the transport of vesicles which pitched off from the plasma membrane facing compartment one, follow a transcytosis path. In this case, vesicles traffic not to final intracellular destinations but across the cell body, which in some instances happens through intermediate compartments, and finally exocytose and fuse with the opposite plasmalemma to release cargo in compartment two [33,83]. Because of this property, this trafficking route holds interest in the context of drug delivery across cellular barriers, such as the case of the epithelial lining separating the gastrointestinal tract from the circulation, or the endothelial lining separating the circulation from certain subjacent tissues, for instance, the blood–brain barrier between said circulation and the central nervous system [33,83].

Two main mechanisms have been classically associated with transcytosis transport, the clathrin- and the caveolae-mediated pathways described in Section 2.1 [83,84]. Their relative contribution to this process is unclear, yet some differences have been observed. For instance, in the case of the brain endothelium the clathrin route seems more prominent, while in organs such as the lung the caveolae-mediated pathway is more abundant [83,84]. Examples of caveolae-mediated transcytosis include those which associate with albumin receptor gp60 or aminopeptidase P, while those which associate with the transferrin or insulin receptors are mediated via the clathrin route [38,85,86,87]. More recently, the CAM pathway has also been associated to transcytosis of drug delivery vehicles, both across epithelial and endothelial linings [88,89,90], which resulted in four- to seven-fold enhanced accumulation of therapeutic enzymes in the brain upon intravenous administration of polymer nanoparticles [91,92].

## 3. Alterations in the Vesicular Transport of Cells

### 3.1. Diseases Affecting Vesicular Trafficking

**Figure 3:**
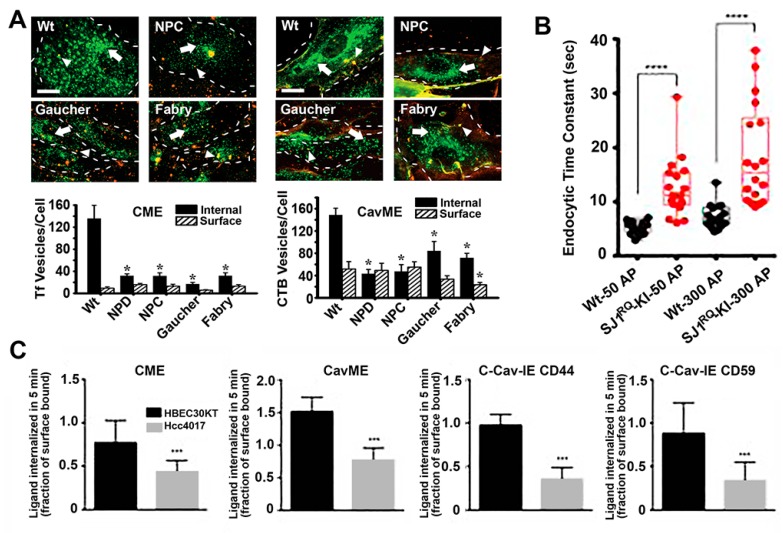
Endocytic alterations caused by disease. (**A**) Micrographs (top panels) and image quantification (bottom graphs) of the uptake of transferrin (Tf; left side) via clathrin-mediated endocytosis (CME) or cholera toxin B (CTB; right side) via caveolae-mediated endocytosis (cavME) in fibroblasts from wild-type (Wt) individuals or patients of Niemann–Pick type A (NPD), Niemann–Pick type C (NPC), Gaucher and Fabry diseases. Green: internalized ligand; yellow-red:cell surface-bound ligand; dashed lines: cell borders; scale bar: 10 μm. * *p* < 0.05, Student’s *t* test. (**B**) Endocytosis time constants after short (50 action potentials) or long (300 action potentials) stimulation of cortical neurons from Wt mice vs. mice expressing Parkinson-like R258Q mutation in synaptojanin 1 (SJ1), a molecule involved in synapse endocytic signaling. **** *p* < 0.00001, Mann–Whitney *U* test. (**C**) Uptake of endocytic markers in HBEC30KT normal cells vs. Hcc4017 cancer cells from the same patient. C-Cav-IE: Clathrin- and caveolae-independent endocytosis. *** *p* < 0.0005, Student’s *t* test. Data are mean ± standard error of the mean (SEM) for (A) and (B), and standard deviation (SD) for (C). Adapted and reproduced with permission from: (**A**) Figures 4 and 5 in [94]; (**B**) Figure 5B in [95]. Copyright 2017 Elsevier Inc.; (**C**) Figure 2A in [96]. Copyright 2015 American Association for Cancer Research.

As described in the Section 2, the molecular machinery and regulation of cellular processes involving vesicular trafficking are highly complex. Because of this and because these processes are intimately interconnected with a broad number of cellular functions, vesicular transport is susceptible to alterations in many disease conditions (Figure 3). For instance, neurodegenerative disorders such as Alzheimer’s or Parkinson’s diseases, autoimmune diseases and many cancers have been linked to alterations in endocytic transport, lysosomal processing, autophagy, etc. [19,20,93]. In most cases, these dysfunctions associate with aberrant accumulation of undigested substances in both lysosomal and autophagic compartments within cells, which typically leads to nutrient deprivation, altered signaling and/or metabolism, apoptosis and inflammatory phenotype, which ultimately contributes to disease progression [93].

#### 3.1.1. Lysosomal Disorders

Lysosomal disorders (LDs) are the clearest clinical illustration of these effects. This is a group of ≈60 different conditions affecting humans and animals, which are due to inherited mutations in genes that encode for lysosomal components (enzymes, transporters, cofactors, etc.) or molecules involved in their biogenesis [97]. As a consequence, lysosomal functions become affected, which results in aberrant accumulation of undegraded materials within this compartment [98]. This primarily impacts the metabolic balance in cells and secondarily alters intracellular trafficking. This is because said lysosomal storage and dysfunction affects the display of lysosomal transmembrane proteins, their interaction with cytosolic molecules and their interaction with pre-lysosomal compartments such as the case for autophagosomes and endosomes [93,99]. In addition, these lysosomal alterations also affect the secretory pathway, recruitment of cytoskeletal elements and vesicular transport [93,99]. Hence, the pathological phenotype of LDs arises not only from the originating genetic/biochemical defect but also from all these secondary disbalances [93,99]. Ultimately, because lysosomes are ubiquitous in cells through the body, LDs alter numerous tissues, including peripheral organs and the central nervous system, and often results in premature mortality [100].

Many examples of alterations in vesicular transport have been observed in these syndromes. For instance, our group investigated the status of fluid-phase and receptor-mediated endocytosis in fibroblasts from patients of four different LDs, including acid sphingomyelinase deficient Niemann–Pick A disease, NPC1 transporter deficient Niemann–Pick C disease, α-galactosidase A deficient Fabry disease and glucocerebrosidase deficient Gaucher disease [94]. We observed that most endocytic pathways were altered, although at different extents in different disease types, including clathrin- and caveolae-mediated endocytosis of ligands and fluid-phase and macropinocytosis [94]. Similarly, others identified alterations in clathrin-dependent recycling of synaptic neurotransmitters in Gaucher and Batten disease, which may contribute to some of the neurological symptoms observed in these diseases [101,102]. Alterations in the caveolar route associated with LDs characterized by lipidosis, such as the case for Niemann–Pick A, Niemann–Pick C and Gaucher diseases investigated in our study, as well as Batten and Krabbe diseases where others had observed disruption and poor recruitment of signaling molecules to lipid rafts domains [103,104]. Many other groups have observed alterations in the behavior of endocytic receptors and pathways in LDs [101,103,105,106,107,108].

Importantly, not all routes were affected in all LDs studied and some were either diminished or enhanced depending on the particular disease. This was the case for macropinocytosis, which was inhibited in Niemann–Pick type A but enhanced in Niemann–Pick type C [94]. This result may be due to the fact both clathrin and caveolar routes were lowered in Niemann–Pick type C cells, for which they may enhance macropinocytosis as a means to cope with the endocytic function. Although this would also be expected for Niemann–Pick type A, the enzyme deficient in this disease is involved in the signaling cascade regulating macropinocytosis [109], hence the inhibition of this pathway. Also, interestingly, because the alterations observed in the caveolar route were less acute in Gaucher and Fabry cells, this pathway was somewhat active and compensatory mechanism were less needed, which may explain why macropinocytosis was not altered in these disorders.

In addition to endocytic uptake, other cellular functions depending on vesicular transport, fusion, etc., are also affected in many diseases. This is the case for autophagy, a process by which cells degrade intracellular molecules and organelles, such as ER fragments, mitochondria, ribonucleic acid (RNA), carbohydrates, soluble proteins, lipids and so forth. [110]. In the macroautophagy route, the components that need to be digested are first separated from the cytosol by formation a double-membrane vesicle termed autophagosomes [111]. These compartments then fuse with early and late endosomes and finally with lysosomes, which enables the degradation of the contained materials [111]. This is regulated by autophagy-related genes (ATGs), mammalian target of rapamycin (mTOR) kinase and beclin-1/phosphatidylinositol 3-kinase (PI3K) type III complex [110]. Many LDs, such as ompe disease, mucolipidosis IV, mucopolysaccharidosis IIIA and others, have been shown to be associated with a lower rate of fusion between autophagosomes and lysosomes, which causes retention of materials in autophagic vesicles [112,113]. In some cases, this appears to be due altered distribution of SNAREs in cholesterol-enriched domains in the membrane of lysosomes and autophagolysosomes [114]. Failure of autolysosome clearance and mTOR reactivation has also been seen for some LDs [115].

#### 3.1.2. Alzheimer’s Disease

Importantly, the case of LDs is not unique. Autophagic and endocytic alterations have also been observed in many other diseases. This is the case for Alzheimer’s. This common neurodegenerative disorder associates with accumulation of extracellular senile plaques of β-amyloid (Aβ) and intracellular phosphorylated Tau-containing neurofibrillary tangles in the brain [116]. In this disease, lysosomal accumulation of undigested substrates and dysfunction of the autophagic pathway are also encountered [116]. This is in part due to the presence of mutated presenilin-1 (PS1), which alters the glycosylation and trafficking of the vacuolar H^+^-ATPase, resulting in poor activation of lysosomal hydrolases [116]. In addition, mutated amyloid precursor protein (APP) leads to accumulation of Aβ peptides and ubiquitinated proteins, which lowers the autophagic flux and impairs both endolysosomal vesicles and autophagosomes [116]. Therefore, these similarities with LDs would suggest that endocytic processes may also be altered in Alzheimer’s. In fact, there is sufficient evidence demonstrating this. For instance, overactivation of Rab5 has been recently shown in postmortem brain samples of Alzheimer’s patients, which has been associated to alterations in Ras and Rab interactor (3RIN3), a guanine nucleotide exchange factor for Rab5 [20]. In addition, other Alzheimer’s associated loci include: (i) the phosphatidylinositol binding clathrin assembly protein (PICALM), whose expression is reduced in this disease [117]; (ii) bridging integrator 1 (BIN1), an adaptor protein of the clathrin-mediated pathway, which is increased in Alzheimer’s [118]; (iii) sortilin-related receptor 1 (SORL1), which regulates intracellular trafficking and processing of clathrin adaptors [119]; and many other [20], which suggests that altered endolysosomal processes contribute to the pathogenesis of this disease.

#### 3.1.3. Huntington’s Disease

Lysosomal dysfunction, impaired autophagy and endocytic defects are also found in Huntington’s. This is an inherited disease affecting huntingtin (htt) protein [120]. Mutant htt activates both the endolysosomal route and also autophagy, resulting in enhanced lysosome numbers and tubulation of endosomal membranes [121]. Autophagosome turnover is also lowered in this disease and, as a consequence, autophagosomes accumulate in the cytosol and the perinuclear region of the cell [121]. Both clathrin-mediated and caveolae-mediated endocytosis have been observed to be altered in Huntington’s [122,123]. For instance, aggregation of disease-associated proteins inhibits endocytosis of membrane receptors associated to neuronal function, which occurs via aggregate-mediated sequestration of the molecular chaperone heat shock cognate protein 70, required for this pathway [122]. In addition, mutant htt was seen to interact with caevolin-1, which causes high accumulation of plasmalemma cholesterol and lowered endocytosis of the membrane lipid lactosylceramide [123].

#### 3.1.4. Parkinson’s Disease

Parkinson’s, which is caused by the aggregation of the cytosolic protein α-synuclein in neural tissue, also associates with lysosomal, autophagy and endocytic dysfunctions [124]. For instance, mutated forms of α-synuclein cannot be properly degraded in this disease because of high affinity to the lysosomal protein LAMP-2A and this also alters ubiquitin-terminal esterase L1 in patients [125]. As a consequence, mutated α-synuclein and other materials are aberrantly accumulated in Lewy bodies and there is also accumulation of large secondary lysosomes containing lipofuscin [125]. Recently, it has been shown that a homozygous mutation in the Sac domain of synaptojanin 1, a phosphatase involved in the signaling cascade regulating endocytosis of synaptic vesicles in neurons, associates with early-onset Parkinson’s [95]. This mutation causes endocytic defects and a massive accumulation of clathrin-coated intermediates and dystrophic axonal changes in dopaminergic axons, showing the connection between synaptic endocytic dysfunction and Parkinson’s [125].

#### 3.1.5. Cancer

Different types of cancers also associate with alterations in endocytosis, autophagy and vesicular trafficking [19,126]. For instance, established tumors generally have a high metabolic demand, with most internal tumor areas being exposed to relatively hypoxic conditions [127]. To overcome this, cancer cells typically regulate autophagy to promote tolerance and survival, which is mediated, among other molecules, by beclin-1, a regulator of autophagy that appears deficient in 40–75% of human breast, prostate and ovarian cancers [128]. In addition, many other abnormalities related to vesicular transport have been observed as hallmark of cancer and malignant transformation, such as altered casitas B-lineage lymphoma (Cbl) and neural precursor cell-expressed developmentally down-regulated protein 4 (Nedd4). These are ubiquitin ligases which regulate endocytosis of some molecules or mark them for proteasome degradation. Therefore, their alteration in cancer generally prevent removal of molecules which should otherwise be downregulated or removed to maintain cell normal homeostasis [129]. In addition, cancer cells present unbalanced recycling and regulation of growth factor receptors, integrins and junctional proteins, abnormal cytoskeleton interactions and alterations of Rabs and so forth [19]. For instance, signaling and protein complexes involved in cell adhesion to the substrate and neighboring cells are dynamically assembled/disassembled and either recycled or trafficked to lysosomes for degradation through vesicular transport. Tumor-initiating and metastatic cells alter this balance, contributing to cancer initiation and progression [19]. Similarly, by reducing endocytosis of growth factors receptors, cancer cells enhance their response potential to said growth factors, supporting their cancer phonotype [19]. In particular, a study examining the endocytic activity of syngeneic models for normal and oncogene-transformed human lung cells showed decreased endocytosis by clathrin-mediated, caveolae-mediated and independent pathways [96]. However, these observations may differ depending on the particular cancer phenotype, e.g., the clathrin coat adaptor huntingtin-interacting protein 1 (HIP1) seems to be overexpressed in some epithelial cancers and certain mutant forms of hepatocyte growth factor receptor (HGFR) display increased endocytosis, which contributes to tumor progression [130]. Many cancers associate with caveolin-1 downregulation and yet others do with upregulation of this marker [131].

#### 3.1.6. Lipidoses

Finally, diseases which cause significant changes in the lipid composition and distribution in cell membranes are also hot targets for endocytic and vesicular trafficking alterations. This is because the lipid profile of membranes along the endolysosomal pathway has a major relevance in the regulation of the physicochemical and biological properties of the said membranes, which in turn regulates vesicular formation, trafficking, fusion, etc. For instance, formation of lipid–protein complexes may influence the generation of membrane curvature and the sorting of membrane-bound material highly depend upon the lipid composition [106,107]. Lipids located in the extracellular leaflet of the plasmalemma or the lumen of intracellular vesicles, such as sphingolipids, are known to regulate the activity of ion channels, which is involved in exocytosis and membrane remodeling [132,133]. Increased levels of cholesterol have been shown to alter the distribution and function of SNAREs complexes and this impacts vesicular fusion with acceptor membranes [114].

Therefore, alterations of vesicular transport routes are rather common in many and diverse diseases, although the specific pathway and function affected varies among them and needs to be studied for each case. Nevertheless, this commonly overlooked aspect is of paramount relevance in terms of selecting and designing drug delivery means which can capitalize vesicular routes active (not halted) in each particular condition. An example of said relevance is illustrated in the case of treatment of LDs by therapeutic enzymes, which require endocytic uptake and lysosomal transport for effective treatment. For instance, in the case of treatment of Niemann–Pick A and Pompe disease, it has been reported that, because of the endocytic alterations in these diseases, intracellular delivery of therapeutic enzymes was suboptimal [134,135]. However, bypassing the defective clathrin-mediated route by targeting the same therapeutic enzymes to the CAM pathway, which was not observed to be defective in these diseases, efficient delivery and effects were achieved [136,137,138].

### 3.2. Therapeutic Drugs and Supplements Impacting Intracellular Sorting

**Figure 4:**
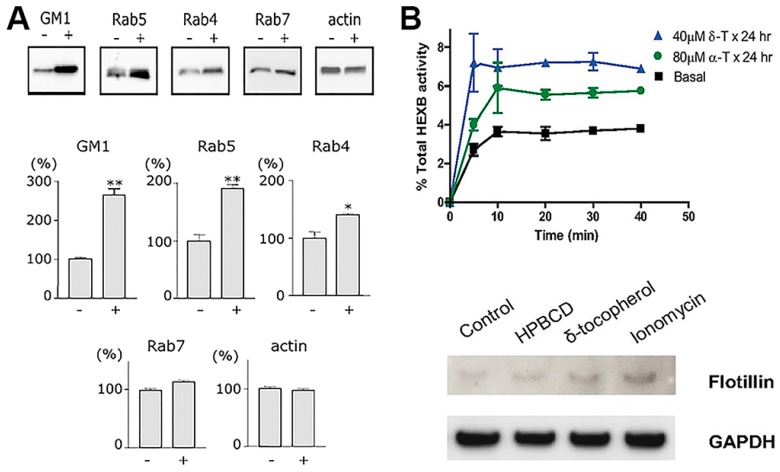
Endocytic alterations caused by therapeutic drugs. (**A**) Western blot protein bands (upper panels) and densitometry (bottom panels) showing the effect of the antimalarial and cancer treatment drug, chloroquine, on the level of vesicular transport elements in PC12 cells activated for endocytosis with cholera toxin B. (**B**) The upper graph shows lysosomal exocytosis, measured as extracellular release of lysosomal enzyme HEXB, in fibroblasts from normal individuals treated with by δ- or α-tocopherol. The bottom panel shows Western blot analysis of flotillin-2 in exosomal fraction of cell treated with hydroxypropyl-β-cyclodextrin (a positive control), δ-tocopherol, or ionomycin, normalized by glyceraldehyde 3-phosphate dehydrogenase (GAPDH) levels. Data are mean ± SEM, ** *p* < 0.05, Student’s *t* test. Adapted and reproduced with permission from: (**A**) Figure 2 in [139]. Copyright 2006 Federation of European Biochemical Societies; (**B**) Figure4 in [140]. Copyright 2012 The American Society for Biochemistry and Molecular Biology, Inc.

Many pharmaceuticals utilized for disease treatment act upon cell surface receptors and/or intracellular machinery which are involved in signaling and trafficking relative to the vesicular pathways described in previous sections. In fact, it is precisely because of this action that said pharmaceutical agents are also utilized to study receptor-mediated and fluid-phase endocytosis and trafficking of cargo. Therefore, in the realm of drug delivery, one must take into account the potential side effects that a drug cargo may have on said endocytic and trafficking processes, as this may in turn alter and impact the efficacy of intracellular drug delivery. Basically, while advantageous to treat the disease for which they were designed, drugs may also secondarily lower access into cells, resulting in efficacies which are unpredictably lower than expected (Figure 4 and Table 2). Surprisingly, this is commonly overlooked and may very well contribute, among other factors, to explain drug resistance phenomena.

#### 3.2.1. Drugs That Affect the Cytoskeleton

Most obvious cases of pharmaceuticals which alter vesicular trafficking are those relative to compounds which affect the cytoskeleton. For instance, many antineoplasic agents exert their activity by interacting with tubulin and inhibiting dynamic polymerization of microtubules required for cell division [141]. Since microtubules are also necessary for intracellular trafficking of intracellular vesicles, some of these agents are commonly used to investigate which particular pathway depends or not on this cytoskeletal element. Hence, these drugs may not be amenable for delivery optimization using drug carriers which depend on vesicular trafficking to access cells. This is the case for agents such as vinca alkaloids (vinblastine, vincristine, vindesine, etc.), nocodazole, colcemid, colchicine and so forth. As an example, colchicine, is a natural metabolite from plants which has been used clinically in the treatment of gout, familial Mediterranean fever, Behçet’s disease, among others [141]. This is due to its inhibitory activity on the motility and secretion from neutrophils, which renders anti-inflammatory effects useful to patients who cannot tolerate long-term use of other anti-inflammatory agents [141,142]. Vinblastine, another example, is a chemotherapy employed with other medications for a broad number of cancers, such as non-small lung cancer, melanoma, testicular cancer, and Hodgkin’s lymphoma [143]. It promotes cell arrest at the M phase due to its inhibiting action on the formation of the mitotic spindle and kinetochore. This agent, as well as nocodazole or colcemid, seems to have different modes of action depending on the concentration. At low concentrations, it binds to the plus-end of microtubules and inhibits their dynamics, while at high concentration it lowers microtubule polymer mass, apparently mediated by action on the minus-end of microtubules, which leads to microtubule detachment from the organizing center, fragmentation, etc. [144]. Although indirect, several examples indicate that, in fact, secondary effects of these drugs relate to their action on microtubules involved in vesicular trafficking and the resulting homeostasis among the intracellular compartments which depend on these processes. For instance, it was long observed, in rats which had been treated with vinblastine or colchicine, that fusion between nascent autophagosomes and secondary lysosomes was enhanced in liver cells, as well as the number of lysosomes in cells of the bile canaliculi, with additional decrease in the secretion of enzymes [145,146].

#### 3.2.2. Lysososomotropic Agents

Additional to drugs that directly impact the cytoskeleton, those which accumulate in lysosomes (lysosomotropic agents) and/or alter the pH of intracellular vesicular compartment. These agents diffuse through cell membranes at neutral pH but accumulate within lysosomes because their protonation within this compartment renders them unable to escape these vesicles [21]. They represent examples with potential to hinder intracellular drug delivery. For instance, Resochin^®^ and Dawaquin^®^ are drugs whose active principle is chloroquine, an aminoquinoline mild-base which accumulates in lysosomal compartments of cells. Chloroquine acts as a buffer avoiding lysosomal acidification and activation of lysosomal compartments, and secondary impacting trafficking and other functions of this compartment. In the clinics, chloroquine has been long used as an antimalarial drug owing to its capacity to inhibit the heme polymerase enzyme, which is present in the parasite trophozoite digestive vacuole while in erythrocytes [147]. Chloroquine has also been tested in clinical trials as an antiretroviral agent against HIV/AIDS [148] and as a chemosensitizing compound in several anticancer trials. This is because it affects formation of autophagic vacuoles, where it has been observed to impact both cancer cells and other cells present in the tumor environment [149]. This effect associates with alterations in vesicular formation and trafficking and other effects have also been observed. Some of them are the lowering of antigen presentation in dendritic cells, reduced secretion of lysosomal enzymes and diminished secretion of reactive oxygen species and so forth, which have been studied for anti-inflammatory purposes, such as treatment of rheumatoid arthritis [21]. In addition, alterations in the lipid content of Rab5-positive early endosomes have been observed, with concomitant endosomal enlargement [139]. Chloroquine-mediated impact on the endosomal trafficking of Notch1, as well as formation of signaling platforms in endothelial cells, are effects that contribute to increasing the quiescent phenotype of the endothelial cells in some tumor settings [150]. Chloroquine has also been observed to alter the trafficking and fusion of glutamate transporter 4 (GLUT4) with the plasmalemma and to inhibit endolysosomal degradation of insulin (and other molecules, as expected), which cause insulin–insulin receptor accumulation in endosomes, for which this drug has also been studied in the context of diabetes [151,152].

Other lysosomotropic compounds have been applied in the clinics. For instance, Cordarone^®^ or Nexterone^®^ are commercial formulations of the active principle amiodarone, used in the context of arrhythmia. This compound lysosomotropic properties render numerous sides effects and hepatotoxicity, such as lysosomal engorgement and storage-like disease in several organs including the lungs, brain, skin, etc. [153,154,155,156,157,158]. Suramin is another lysosomotropic medication which is used for the treatment of African sleeping sickness and River blindness due to parasitic infection [159]. The drug accumulates in lysosomes in the liver, kidney and spleen, most predominantly in Kupffer cells and macrophages [160,161], inducing lysosomal storage and associated vesicular transport defects [162].

#### 3.2.3. Aminoglycosides Antibiotics

Compounds such as streptomycin, kanamycin, gentamicyn and otehrs, are additional examples of drug affecting intracellular vesicular processes. These compounds act on Gram-negative bacteria, for example, *Pseudomonas*, *Enterobacter*, *Acinetobacter*, as well as Mycobacteria, by inhibiting protein synthesis and somewhat affecting also the functional integrity of the bacterial cell membrane [163]. Effects of these drugs have been observed in kidney lysosomes, although the mechanism was obscure [21]. In addition, trospectomycin sulfate and azithromycin showed trafficking defects leading to the formation of intracellular “lamellar bodies” in kidney, spleen, liver, myocardial blood vesicles and so forth, in large animal models [164,165].

#### 3.2.4. Lipophilic Vitamins

Several derivatives of vitamin E appear to be capable of insertion within the plasmalemma of cells, resulting in changing the its bending curvature [166]. Although not much research is available in the literature in this regard, this has been speculated to have an impact on the display and perhaps function of membrane receptors in the vicinity, as well as endocytic events or other vesicular trafficking processes [167]. In fact, several studies have shown that δ-tocopherol profoundly enhanced exocytosis of lysosomes and, likely, endosomes [140]. Precisely, in virtue of this property, this vitamin is being investigated as a potential therapeutic avenue to lower pathological lysosomal storage of undegraded molecules [140]. However, as said, it is possible that this activity also alters endocytic transport, as preliminary results in our laboratory strongly suggest: cells treated with δ-tocopherol and several derivatives displayed lower activity for both pinocytic and receptor-mediated processes, although such effects were transient and trafficking was restored several hours after treatment. This can be explained based on the fact that tocopherols have been shown to disrupt plasmalemma recruitment of protein kinase C (PKC) [6,168], which is signaling molecule involved in all most common uptake pathways, including clathrin- and caveolae-mediated endocytosis, phagocytosis and the CAM pathway.

### 3.3. Impact of Drug Carriers in Vesicular Function

Drug delivery strategies are those aimed to improve the bioavailability of drugs by modulating their solubility, stability, circulation, targeting and biodistribution, subcellular transport, and/or release rate [169,170,171,172,173,174]. Therefore, these approaches help enhance the therapeutic value of drugs and minimize their toxicity [169,170,171,172,173,174]. A variety of formulations have been designed for this purpose, from conjugates to nanoparticle carriers, including linear, branched and dendrimeric polymers, micelles, liposomes and polymersomes, several types of porous and solid particles, and modified versions of natural vesicles [172,173,174,175,176,177,178]. These carriers can be fabricated out of natural and/or synthetic materials, organic and/or inorganic ones, and may be biodegradable at different extents and through different mechanisms [172,173,174,175,176,177,178]. Their performance and functionality can be adjusted by tuning their physical and chemical features [11,12,13,14,15,16,177,178,179] and they can be additionally coupled to moieties which facilitate their interaction with and transport by cells in the body [4]. For instance, these systems can be attached to positively-charged peptides, such as the HIV Tat peptide or other cell penetrating motives, or their polymeric counterpart may be itself positively-charged, which facilitates interaction with non-specific elements of the plasmalemma such as negatively-charged glycoproteins [170,178]. Other affinity moieties include natural ligands, antibodies, peptides, vitamins, aptamers and other elements which can be selected to recognize and bind to specific cell-surface receptors involved in uptake and vesicular transport [33,85]. By specifically binding to said receptors, carriers and their drug cargoes can be mobilized across cellular monolayers by transcytosis, or taken inside cells by endocytosis, followed by subcellular transport to several compartments [3,4,5] (see Section 2.3 above). In general, these targeting strategies improve the biodistribution of drugs, although their success depends on the accessibility of the target tissues from the administration site and their ability to cross biological barriers in the body [3,4,5].

#### 3.3.1. Effect of Carrier Geometry, Mechanical and Surface Properties

Parameters such as the size and shape of drug carriers, targeting valency, surface charge or “fouling” properties, or their biomechanical features are factors known to influence their own cellular uptake and trafficking. Many articles offer outstanding discussions on these topics [11,12,13,14,15,16,17,18,180,181,182]. Only a summary is presented here, since the main goal of this section is to focus on how drug carriers affect endogenous (not just their own) endocytosis, which is discussed in Section 3.3.2 and Section 3.3.3 below.

**Figure 5:**
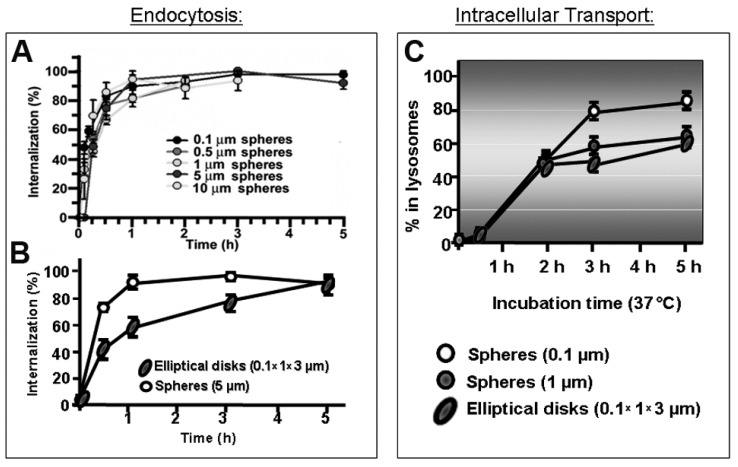
Role of carrier size and shape in the cellular uptake and trafficking via intercellular adhesion molecule 1 (ICAM-1). (**A**) Kinetics of endocytosis of spherical, ICAM-1-targeted polymer particles of various sizes by endothelial cells in culture. (**B**) Kinetics of uptake of micrometer-range size, ICAM-1-targeted polymer particles of spherical vs. elongated-disc shape. (**C**) Kinetics of lysosomal trafficking of said spherical vs. elongated polymer particles, also comparing nano- vs. micrometer-size range. Data are mean ± SEM. Adapted and reproduced with permission from Figures 4B and 6B in [184]. Copyright 2008 The American Society of Gene Therapy.

Given natural constraints of the size of membranous vesicles which can form at the plasma membrane (Section 2.1 and Table 1), the geometry of drug carriers impacts their cell uptake and trafficking [11,15,17]. Clathrin- and caveolae-dependent pathways operate with highest efficacy below 50–150 nm in diameter, for which drug carriers over this range display decreased transport, such for transferrin receptor- or aminopeptidase P-targeted systems [9,38,183]. Instead, the CAM pathway is less impacted by carrier size, where efficient endocytosis of micrometer-range carriers was observed in cell cultures and in vivo [184,185], although lysosomal trafficking was delayed by increasing particle size (Figure 5). This “flexibility” is due to a built-in mechanism which regulates the sphingomyelin–ceramide content of the plasmalemma: carriers targeting the CAM pathway induce cellular secretion of sphingomyelinases at carrier-binding sites [185]. In consequence, coupling sphingomyelinases on the surface of drug carriers which targeted non-CAM pathways provided a means to similarly control said sphingomyelin–ceramide balance. This strategy enhanced endocytosis of micrometer-size carrier targeted to clathrin-dependent receptors [13]. Noticeably, depending on the therapeutic cargo and pathology to be treated, the uptake efficacy may not be the most important parameter in achieving the desired effect. For instance, the antioxidant effect of superoxide dismutase in endotoxin-challenged endothelial cells was more effective when delivered via caveoli vs. CAM, despite a lower uptake of the former route. This is because the oxidative species which endotoxin induced in these cells had been produced within the caveolae-associated endosomes which received the antioxidant enzyme [186]. Also interestingly, in the case of targeting caveolae-associated PLVAP, substituting rigid polymer carriers which showed poor access to the plasmalemma by more deformable gel counterparts, targeting and lung uptake was considerably enhanced even without lowering the carrier size [10]. However, biomechanical properties of drug carriers influence uptake in ways still not fully understood [12]: rigid lipid-coated poly(lactide-co-glycolide) (PLGA) nanoparticles are more efficiently internalized by tumor cells than their softer counterparts [187]. Other works showed a sift between membrane fusion and endocytic uptake depending on the particle elasticity [188].

With regards to particle shape, numerous studies have shown the profound influence of this parameter in vesicular transport. For example, despite the flexibility of the CAM pathway for uptake of micrometer-range carriers, elongated particles showed reduced uptake efficiency compared to spherical counterparts [184] (Figure 5). Amorphous conjugates caused incongruent receptor clustering, with several independently engaged patches of the receptor per conjugate particle, which lead to less effective internalization [189]. For non-spherical, high aspect ratio particles investigated in phagocytic cells, the angle of contact with the plasmalemma plays a relevant role in the particle uptake efficacy: phagocytosis was reduced and even halted at areas of low curvature [12].

Surface properties of drug carriers additionally modulate these outcomes. It is well known that increasing a carrier targeting valency (the number of affinity ligands displayed on the drug carrier surface) can enhance binding and uptake by cells up to a “receptor-saturating” level [190]. Interestingly, it is not the absolute number of affinity moieties that engages receptor molecules on the cell surface which regulates signaling and uptake induced by the carrier but the “valency density,” which is the number of affinity moieties engaged per surface area [190]. Interestingly, valency changes do not affect the mechanism of uptake in some instances, for example, CAM-mediated uptake of ICAM-targeted carriers [190]. Conversely, valency changes affect uptake mechanism in other cases, such as for folate receptor-targeted quantum dots (QDs), which switched from caveolae-mediated uptake at low valency to clathrin-dependent one at high valency [191]. In addition, deposition of serum proteins on a drug carrier surface, which is influenced by the particle surface charge, further modulates cellular interactions [181], although this has been mainly characterized using immune cells and non-targeted nanoparticles. The use poly(ethylene glycol) (PEG) to minimize these non-specific interactions, which unfortunately has a similar effect on specific ones, has been extensively discussed [192]. Finally, surface coating of the “don’t eat me” signal provided by cluster of differentiation (CD) 47 on drug carriers has been shown to lower phagocytic internalization [193] without affecting specific binding and uptake by co-coated targeting moieties [194].

The still low number of systematic studies using unified models (carriers, cargo, cell or animal models, diseases, etc.) [12] makes it difficult to obtain generic conclusions, for which theoretical studies can help interpret and, perhaps, predict drug carrier behavior [195].

**Figure 6:**
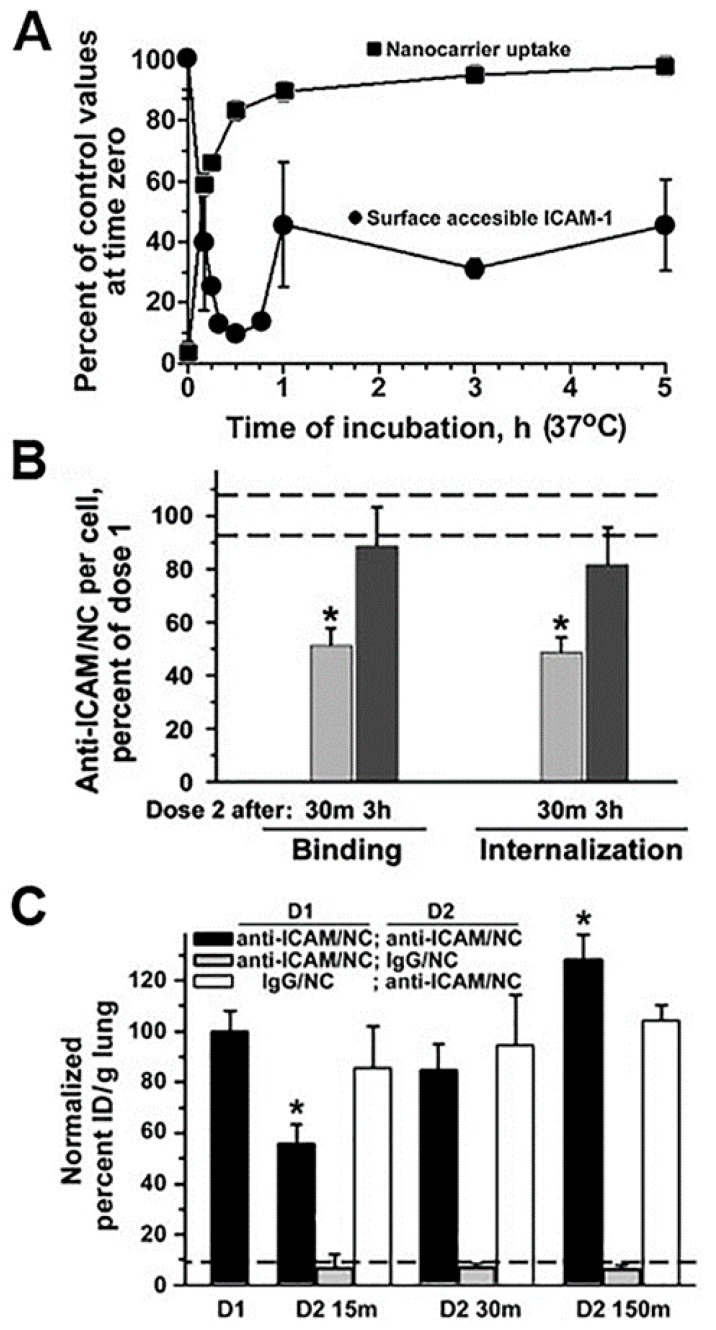
Endocytic alterations caused by drug carriers. (**A**) Kinetics of endocytosis of via intercellular adhesion molecule 1 (ICAM-1)-targeted polymer nanocarriers (anti-ICAM/NCs) by HUVEC cells (traced by fluorescence microscopy) and that of cell surface levels of ICAM-1 receptor during nanocarrier uptake (traced by radioactive labeling). (**B**) Relative level of binding and endocytosis of anti-ICAM/NCs applied as a second dose to HUVEC cells (and traced by fluorescence microscopy), either 30 min or 3 h after a first dose of NCs. * *p* < 0.05, Student’s *t* test. (**C**) Relative level of binding and endocytosis of anti-ICAM/NCs applied as a second dose to HUVEC cells (and traced by fluorescence microscopy), either 30 min or 3 h after a first dose of NCs. (**C**) Lung targeting, expressed as % injected dose per gram of tissue (%ID/g; radioactive tracing), of a first dose of anti-ICAM/NCs injected i.v. in mice vs. that of NCs applied as a second dose 15, 30, or 150 min after the first dose. Control non-specific IgG/NCs are also shown. Data are mean ± SEM, compared by Student’s *t* test. Adapted and reproduced with permission from Figures 1 and 3 in [64]. Copyright 2005 The American Sociaty of Hematology.

In addition to these well-studied factors which influence the uptake efficiency, mechanism and destination of drug carriers, a carrier “side effects” on the endogenous vesicular transport processes are largely unknown and overlooked. Whether passively or via active targeting, drug carriers possess the ability to enter cells in the body, both at the intended locations and at body sites involved in clearance of foreign materials (the liver, spleen, lymph nodes, etc.) [4]. Tissue cells as well as cells of the immune system internalize these systems via vesicular trafficking, whereby their most common intracellular destination is the endolysosomal route, as described in Section 2.3 above [93]. Therefore, it is highly likely that drug carriers interfere with the natural use of said vesicular trafficking pathways and their accumulation within cells may cause alterations of these pathways [22,196]. The following subdivisions within Section 3.3 highlight this aspect (Figure 6).

#### 3.3.2. Role of Carrier Biodegradability

Carriers which contain non- or less readily-degradable materials are known to accumulate within vesicular compartments within cells and have been seen to alter the lysosomal/autophagic balance [197,198]. This has been studied for fullerenes and graphene nanoparticles, QDs and metal-containing formulations and dendrimers which appear to activate autophagy [199,200]. Numerous reports show the presence of nanoparticles in double-membrane vesicles consistent with autophagosomes and their ubiquitination, which is known to be involved in the process of transport to autophagosomes by p62 [201]. Therefore, this may represent a rather generic cellular defense reaction to nanoparticle exposure [22]. Excessive induction of autophagy can result in the accumulation of autophagosomes, which is detrimental for disease progression. In addition, prolonged “storage” of these materials within lysosomes in cells has been observed, which reduces the autophagic flux and results in autophagosome accumulation [22].

Importantly, these effects are not exclusive of metal systems or low-degradable materials, but also intracellular accumulation of lipids used in liposomes or other polymers have been associated with unwanted effects, rather reminiscent of lysosomal storage [202,203,204]. This must be understood under the light that drug carriers are used for treatment of disease conditions and, as described in Section 3.1, many of these pathologies compromise per se the regulation of the transporting and degradative machinery of the cell. Therefore, although many materials designed for drug delivery may be biodegradable and negligibly toxic in healthy models, their intracellular degradation and subsequent impact on vesicular trafficking in the disease situation for which they were designed must be carefully investigated. In addition, because of limitations of common cellular models to grow for sustained periods of time, there is lack of information about the effects of recurrent dosing of drug carriers in cell cultures. Some work on this topic identified relevant changes in the case of metal nanoparticles [205] but similar works are needed for most common biodegradable materials. This is of high relevance, since most of the available investigations on this end come from studying single dose administration in cell cultures. Yet, this does not reflect recurrent dosing in vivo and, hence, the compounding effects in the kinetics of intracellular degradation and effects of sustained accumulation in tissue and clearance cells are largely unknown.

#### 3.3.3. Carriers That Cause Endosomal Escape

Particular attention must be payed to systems designed to access the cytosol of the target cells by escape endo-lysosomal compartments, such as cationic polyamidoamine (PAMAM) dendrimers, cationic polystyrene nanoparticles, cationic PLGA nanoparticles, metal nanoparticles, carbon nanotubes and many others [205,206,207,208,209]. These cationic systems buffer the otherwise acidic lysosomal pH and rupture the lysosomal membrane through increasing the osmolarity in these compartments [206]. For non-cationic vehicles, changes in volume which physically destabilize the endolysosome membrane and generation of reactive oxygen species (ROS) has been speculated as the mechanism to achieve lysosomal permeabilization [210]. In the case of drug carriers which are functionalized particular lysosomolytic proteins and peptides, such as those derived from pathogens, these are known to form pores in the membrane of lysosomal compartment [211]. Importantly, in all of these cases, lysosomal permeabilization is known to cause cytosolic release of lysosomal hydrolases, such as cathepsins and other lysosomal components which lead to oxidative stress [212]. These events impact the outer mitochondrial membrane, which in turn exacerbates the generation of ROS and may result in the induction of apoptosis [208].

Unfortunately, there are few specific works examining the effects of drug carrier accumulation and/or lysosomal disruption under the perspective of understanding their impact on endocytosis, transcytosis and intracellular vesicular transport. However, giving that these systems compete against natural ligands (nutrients, hormones, growth factors, etc.) which use the same receptors and pathways, because they induce abundant internalization with consequent “removal” of membrane receptors, associated signaling platform and cytoskeletal elements and because the kinetics for their separation from said receptors and intracellular trafficking and degradation are different from that of natural counterparts, it is highly likely that intracellular delivery of drug carriers alters (transiently or not) these pathways. An example which started to examine these aspects was that of polymer nanoparticles targeted to ICAM-1. In endothelial cells which were activated with tumor necrosis factor α (TNFα) to mimic an inflammatory condition, when a second dose of anti-ICAM nanoparticles was applied 30 min after the first dose of the same particles, their cell binding was reduced by 50% and their endocytosis was lowered by 68%, with respect to the binding and uptake levels observed for the first dose of nanoparticles [64]. This coincided with the fact that the first dose of nanoparticles was very efficiently internalized (about 85% by 30 min) and, it retrieved large amounts of the bound receptor (ICAM-1) into endocytic vesicles (only 15% remained in the cell surface at that time). This explains the reduced binding and uptake of the second dose of nanoparticles applied to the cells within this time frame. Interestingly, binding and uptake recovered to normal levels when the second dose of nanoparticles was applied 3 h after the first dose. This was observed to be due to the fact that by this time, part of the internalized receptor had been recycled back to the plasmalemma (45% was detectable at the cell surface). In addition, lysosomal transport of the second dose of nanoparticles was lowered (by 70% by 3 h), which was due to the fact that lysosomal compartments were occupied with the first dose of nanoparticles applied. Noteworthy, these observations extracted from cell culture models paired well with in vivo results. For instance, because of the high ICAM-1 expression in the lung and the extensive endothelial surface in this organ, anti-ICAM carriers are known to predominantly accumulate specifically in this organ after intravenous injection in mice (e.g., 140% vs. 12% injected dose/g of lung for targeted vs. control nanoparticles). However, when injected 15 min after a first dose, accumulation of the second dose of particles in this organ was reduced by 45% and it recovered to normal values if the second dose was administered 2.5 h after the first one [64]. Although the kinetics of the events in cell culture vs. in vivo were different (expectedly due to other factors occurring in vivo which are not pertinent to the cellular system), the same tendencies had been observed: occupancy of receptors, vesicular compartments and lysosomes by a first dose of anti-ICAM nanoparticles affected the efficacy of the second dose to reach their targets and be internalized. Although these effects were transient, one must consider the fact that only a previous dose of nanoparticles had been applied, while a more recurrent setting could impair these processes further or more chronically.

## 4. The Biological Microenvironment and Vesicular Transport

An additional caveat to studying and manipulating the mechanisms that regulate vesicular transport as means to help drug delivery applications lies in the models available to this end. Unfortunately, using current methodologies, complex mechanistic studies at this level are not readily feasible in animal models, which are rather used for validation of main aspects examined in cell cultures. Cell culture models offer a greater opportunity to investigate these aspects because of the availability of examination methodologies, their tunability and controllability of the conditions and variables investigated. However, most common and simple cellular models, such as established cells lines, two-dimensional (2D) cultures, monocultures, systems which do not include the influence of flow, models lacking the impact of both neighboring or endocrine organs in the body, do not recapitulate key physiological factors which influence vesicular transport in the real physiological realm.

For instance, established or immortalized cells lines are very useful for their increased resistance to culturing conditions and phenotypic persistence over primary cultures. However, they vary from primary cultures in their genotype, phenotype and overall behavior [213]; hence, results obtained in these systems only offer a gross estimation of the real performance of a drug delivery system. As an example, it was demonstrated that certain hepatic cells lines possessed reduced numbers of mitochondria and different use of metabolic pathways vs. primary hepatocytes, which would impact both drug metabolism and effects [213]. Furthermore, studies comparing retinal pigment epithelium-derived cell lines vs. their primary counterparts showed enhanced expression of proteins associated with adhesion, secretion of extracellular matrix and cell migration, as well as decreased expression of proteins involved in cell polarization and altered expression of components of the cytoskeleton, all functions which necessitate or mediate vesicular transport [214]. Also, because cell lines are more resilient, they are often grown in the absence or low concentrations of serum, which is known to augment the endocytic activity as a compensatory response to enhance uptake of needed nutrients and metabolites [215]. In fact, serum-starved cells have been shown to display enhanced uptake of lipoplexes vs. cells cultured in the presence of serum in the medium. This associated with less pronounced cortical actin in serum-starved cells, which would reduce the plasmalemma tension and increase the cell cross-sectional area, exposing a larger surface for internalization and enhancing micropinocytosis, as observed [215].

Apart from this, another method often used to measure endocytosis in cell culture is flow cytometry, which requires detachment of cells from their substrate. This is commonly achieved by incubating adherent cells with trypsin, which proteolytically cleaves integrin–extracellular matrix bonds. Such a treatment can equally cause proteolytic cleavage of cell surface receptors involved in endocytosis and/or their attachment to drug carriers (e.g., those displaying protein ligands, antibodies, or peptides), rendering artefactual results [216]. In addition, cell detachment from their substrate for flow cytometry measurements leads to enhanced endocytosis via cholesterol-rich domains and, likely, caveolae-mediated endocytosis [217]. Display of integrins and other receptors varies in suspension cells and also under this condition the actin cytoskeleton is reorganized from a fibrillar mode typical of adherent cells to a thick subcortical actin, which is likely to alter endocytosis [218]. Therefore, flow cytometry, although helpful, is not a reliable configuration reflective of vesicular transport in cells which are typically adherent in the body, while it may better reflect the status of non-adherent circulating cells.

Similar to the changes observed between cells in suspension vs. adherent, there are major proteomical, morphological, cytoskeletal and functional changes in cells grown as 2D cultures vs. three-dimensional (3D) counterparts [23,219]. Classical 2D cell cultures are readily available, relatively easy to handle and more economical, for which their use is well extended. However, only a few examples in the body display 2D-like configurations, for example, endothelial or epithelial monolayers which are adhered to a basement membrane only through one side, leaving the luminal membrane “free” to interact with ligands and targeted drug carriers and to engage in active vesiculization [4]. Yet, most other cells in the body are “embedded” within tissues and interact with other cells and/or the extracellular matrix through their entire surface [220]. An organization more similar to this one has been achieved using tissue culture techniques involving various methods, such as the case of hydrogels containing cells, decellularized tissues where cells of interest can be then introduced, multicellular spheroids often used to mimic solid tumors, multilayer cell cultures and so forth. [221]. Said 3D organization impacts the efficacy of using endocytic receptors and vesicular trafficking as means for transporting drugs and their carriers into or across cells. For instance, penetration in order to access the plasmalemma of cells embedded within the tissue is the first obstacle encountered [221]. The tortuosity of interstitium, the size of pores in the extracellular matrix, interaction with proteoglycans, etc., precludes carriers from effectively approaching their target cells [222]. But beyond this non-trivial problem, cells arranged in a 3D conformation present different endocytic behavior than in 2D cultures [223]. In cases such as that of solid tumors with a hypoxic core due to restricted diffusion and oxygen consumption by more superficial cell layers, is this hypoxic condition, which can be mimicked in 3D but not 2D cultures, which results in changes pertaining to the display and function of vesicular transport elements, e.g., caveolin-1 endocytosis [131]. In addition, even in the absence of hypoxia, receptor display and signaling changes occurs between 2D and 3D cultures, such as the case of human epidermal growth factor receptors Her2 and Her3 which form heterodimers in 2D cultures while Her2 homodimers are more abundant in 3D conditions [224]. Consequently, ligand binding to these receptors results in phosphoinositide 3-kinase (PI3K)-mediated signaling vs. mitogen-activated protein kinase (MAPK) signaling, respectively [224]. Vesicular trafficking of cell surface markers has also been reported to be slower in 3D vs. 2D cultures, as for the transmembrane glycoprotein podocalyxin, whose Rab transport to and from the cells surface presented different kinetics in these two culture configurations [225]. Also, a different distribution of caveolin-1 has been shown in 2D vs. 3D culture models of neural cells, whereby caveolin-1 appeared to have a more punctate pattern in 2D cultures compared to a more diffuse localization in 3D cultures [226]. This may arguably reflect its reduced engagement with caveolar vesicles either in the plasma membrane or in transit within cells in the second case [226]. In another example, cardiac cells displayed a spread and flattened shape in 2D cultures while the same cells presented a smaller phenotype, with a large network of microtubules, less amount of mitochondria and lower expression of cell junction and actin partners for 2D vs. 3D cultures [227]. Therefore, results in vesicular uptake and transport of drug and their carriers in classical 2D cultures must be taken with caution, as they may not reflect more physiological situations [228].

In this line, additional microenvironmental factors such as the vascular or interstitial flow, are often overlooked when using cellular models. In the case of vascular endothelial cells, the presence and influence of flow on their structure and function are well recognized. The shear stress of the circulating flow represents an important mechanical factor which is sensed by endothelial cells and mediates important adaptation responses, including gen and protein expression, signaling cascades, morphological and cytoskeletal changes and overall endothelial function [229]. Depending on the vascular bed and type of vessel (macro- vs. microvasculature, arteries vs. veins, etc.), the endothelium is exposed to different levels of fluid shear stress and flow patterns, which are influenced by blood viscosity and velocity, as well as geometrical features (vessel diameter, bifurcations, tortuosity, etc.) of the particular vascular tree [230]. This not only influences the chances of a drug carrier to interact effectively with the endothelium for drug delivery to this tissue or for transport across the endothelial lining into subjacent tissues but also numerous aspects pertaining to endocytosis and vesicular trafficking. For instance, endothelial cells which have been adapted to flow display activation of PKC and Rho, molecules involved in endocytic signaling and cytoskeletal remodeling required for these events [231]. The morphology of flow-adapted endothelial cells varies, so that they align to the direction of the flow, which is mediated by formation of actin fibers organized longitudinally or in parallel to said flow [232]. It has been shown that these changes affect the ability of cells to endocytose drug carriers, such as shown in several works investigating intracellular transport of polymer nanoparticles targeted to ICAM-1 and PECAM-1 [233,234]. It was observed that nanoparticle endocytosis was reduced under shear stress conditions when compared to classical static cell cultures [233]. Importantly, a parallelism was found between cell culture and in vivo endothelial endocytosis of these carriers after intravenous administration in mice: microscopic examination revealed more effective uptake of carriers by the endothelium in capillaries where the flow shear stress is low vs. larger vessels where this parameter is enhanced [233]. Other pathways of vesicular trafficking are also likely to be affected in endothelial cells adapted to flow reflecting physiological conditions, since common endocytosis-mediating elements, such as the cytoskeleton, signaling molecules, protein coats, lipid raft domains, etc., have been shown to be differently regulated in static vs. flow conditions [231,232]. Furthermore, the influence of flow is not limited to the endothelial cells in direct contact with the bloodstream but it also applies to cells within tissues through the interstitial flow that bathes tissue cells [235]. This factor affects the transport of communication molecules and metabolites through tissues in the body and influences matrix architecture and permeability, intercellular signaling, cell morphogenesis, gene and protein expression patterns, cell differentiation, etc. [235]. In fact, the effect of anticancer drugs has been shown to the different in tumor models exposed or not fluid mimicking the interstitial flow [236]. Similar to the endothelium, it is believed that tissue cells sense the interstitial flow through mechano-transduction, mediated by adhesion molecules, transmembrane proteins which attach to the extracellular matrix and the cytoskeleton [237]. Therefore, fluid flow is an important parameter influencing elements involved in vesicular trafficking and should be included in more advanced studies examining transport of drug carriers in cellular models.

Importantly, novel means and tools are becoming available to simulate all these different parameters influencing cellular systems, which include the use of microfluidic chips and devices reproducing more physiologically the effect of flow, fluid volume-to-cell ratios, apical–basolateral polarization, 3D architectures, extracellular matrix and even the presence of multiple cell types and organs to recapitulate body regulation [24]. Their fabrication derives from microelectromechanical systems and microfluidics, which enable the use of very small volumes for testing, are compatible with the length-scale of cellular systems and can better mimic the biological microenvironment [24]. Systems of this type have been described to simulate various properties of the gastrointestinal tract, the liver, kidneys, the heart and the lungs [238,239,240]. These devices can encompass microfluidic networks mimicking the circulation and multiple interconnected chambers which represent body organs and may contain cell types or combinations of them, representative of particular body tissues [238,239,240]. Their use in terms of studying pharmacokinetics and pharmacodynamics of drugs is showing interesting results, somewhat more predictable of the in vivo behavior than other cellular models. Because of this, they hold great promise to become amenable systems to study vesicular transport and effects of drugs and their carriers. Nevertheless, they are currently subject to some limitations, including the stability of these systems for mid-term studies, problems with air bubbles generated in their interior, cellular cross-contaminations, difficulties to mimic simultaneously different fluid compositions in different compartments, overgrowth of one vs. another cell type, leakiness, etc. Yet, it is likely that these improvements and other pertaining to their fabrication will enable their spread commercialization for a broader use.

Finally, although outside the scope of this section focused on biological models, it is important to consider that the methods to characterize vesicular trafficking of drug carriers are equally relevant in rendering conclusive and reliable results. For instance, flow cytometry or radioactive tracing offer reliable and quantitative measurement of drug carrier association to cells [241,242]. Yet, they only modestly inform on their uptake since determination of intracellularly located counterparts depend of quenching or elution of cell surface counterparts, which is rarely fully achievable [242]. Various multifluorescence microscopy techniques offer this advantage but in turn, most do not evaluate the entire population and are more prone to investigator-dependent variation [242,243,244]. The use of lipophilic dyes to trace drug carriers often renders artifacts due to partitioning of these dyes but not necessarily carriers, with the plasmalemma and intracellular membrane of cells [245]. Therefore, each available method offers advantages and limitations, for which multipronged studies which validate data through various techniques are preferred.

## 5. Conclusions

In summary, the use of intracellular vesicular trafficking has become an extended experimental strategy to facilitate delivery of therapeutic compounds across cellular linings which separate body compartments, into subjacent tissue cells and to specific intracellular destinations. However, these pathways are tightly regulated through a complex balance of molecular functions acting in concert, which can be the subject to alterations by numerous intrinsic and extrinsic factors. For instance, many diseases, including neurodegenerative disorders, autoimmune diseases, cancers and genetic syndromes, have been observed to associate with alterations in vesicular transport and processing events, including endocytic uptake, cytoskeletal-mediated transport, cargo-processing by lysosomes, autophagy and others. Because of these functions are intimately related with the maintenance of the cellular homeostasis, said alterations lead to secondary effects, affecting cellular metabolism, signaling cascades, oxidative stress, cell death and so forth, ultimately contributing to and/or exacerbating the disease phenotype. Therefore, using these routes for therapeutic delivery requires a meaningful examination of the pathways readily available in each particular disease condition, rather than capitalizing on generic knowledge extracted from situations where the biology of these mechanisms is not compromised. Extrinsic factors pertaining to delivery of therapeutic compounds, which also affect the status and performance of intracellular vesicular trafficking, are the drugs used and drug carriers themselves. For instance, many pharmaceuticals employed in disease treatment exert direct or indirect effects on the molecular elements or functions involved in endocytosis, cytoskeletal networks, endolysosomal transport, etc., such as the case of lysosomotropic agents, some antibiotics, neoplasic compounds and many others. Therefore, it is questionable whether these drugs can be used in conjunction with drug carriers aimed to vesicular pathways, either loaded in carriers or co-administered to the patient, constituting a new and overlooked form of drug resistance or drug interaction. It is also noteworthy the lack of current knowledge on the effects that carriers themselves exert on the cellular machinery and regulation involved in endocytosis and vesicular trafficking. The fact that drug delivery systems occupy and deviate receptors, signaling cascades, cytoskeletal elements and vesicular degradative routes from their biological function, raises concerns about their potential side effects in practical settings requiring their abundant, frequent, or long-term dosing. Finally, the true advantages and caveats derived from the exploitation of vesicular trafficking pathways for drug delivery are beginning to be realized through the use of more “physiological” cellular models, which recapitulate key factors to which cells are exposed in the body, including 3D, multiculture and multiorgan settings, with proper fluid compositions and fluid-to-tissue balances. While detailed and systematic studies are still needed in these directions, the knowledge to come from these efforts will undoubtedly bring the field forward and closer to the successful translational application of drug delivery strategies.

## Figures and Tables

**Table 1:** Endocytic vesicular transport pathways.

Pathway			Cells	Cell Surface Markers and Receptors	Destination	Inhibitors	Ligands	Initial Vesicle Size
Phagocytosis			Mainly immune system cells (e.g., macrophages), but also endothelial and other cells in some instances	Some integrins, scavenger receptors, mannose receptors, Fc receptors	Mainly lysosomal compartments	- Dynamin disruption - Actin disruption (cytochalasin) - Nocodazole ^1^ - H-7 ^2^ - Wortmannin ^3^	Bacteria, opsonized particles, immune complexes	Predominantly 1–10 μm
Macropinocytosis			Mainly immune cells (e.g., antigen-presenting cells), but also inducible in endothelial and epithelial cells, fibroblasts, etc.	Non-receptor mediated, yet binding to EGFR may induce it	Mainly lysosomes, but also recycling pathways	- Amiloride ^4^ - Nocodazole ^1^ - BIM-1 ^2^ - H-7 ^2^ - Staurosporine ^5^ - Wortmannin ^3^	It does not require ligands (fluid-phase uptake), although it can be activated by EGF	Predominantly 1–5 μm
Clathrin-mediated			Most cells in the body	Insulin receptor, LDL receptor, transferrin receptor, receptors of several growth factors, and some adhesion molecules, etc.	Lysosomes and recycling routes in tissue cells, or transcytosis in cellular barriers (e.g., endothelial, epithelial)	- Dynamin disruption - Actin disruption (cytochalasin) - K^+^ depletion - MDC ^6^ - Amantidine ^7^	Insulin, transferrin, LDL, many growth factors, VCAM-1, P/E-selectins, angiotensin converting enzyme, etc.	Most commonly 100–150 nm, but can adapt up to 250 nm
Caveolae-mediated			Most cell types, although reduced in the blood–brain barrier	Ganglioside GM1, aminopeptidases N and P, the albumin-binding receptor gp60, PLVAP, etc.	Lysosomes, the Golgi and rough ER in some cases, but most prominently transcytosis across the endothelium	- Dynamin disruption - Cyclodextrin ^8^ - Filipin - Genistein ^9^	Cholera toxin, albumin, and affinity molecules (peptides, antibodies, etc.) against aminopeptidases N and P, PLVAP, etc.	Most commonly 60–80 nm, but can adapt to larger sizes
Clathrin- and caveolae- independent	Dymanin-dependent	Some IL receptors	Immune cells, endothelium (and other for CAM; see below)	IL2Rβ, IL4Rα, IL15Rα, and some receptors associated with flotillins (and other for CAM; see below)	Recycling, endolysosomal route, and Golgi	- Dynamin disruption - Actin disruption - EIPA ^10^ - Various others depending on receptor	The corresponding cytokines	Varies from 50 to 150 nm
		CAM-mediated	For PECAM-1, endothelial cells; for ICAM-1, most cells (endothelial, epithelial, fibroblasts, astroglia, neuronal, muscle, mesothelial, etc.)	PECAM-1 and ICAM-1	For monomeric ligands, recycling and transcytosis. For multimeric ligands, lysosomal transport (in tissue cells) and transcytosis (in barrier cells)	- Dynamin disruption - Actin disruption (latrunculin) - Amiloride ^4^ - EIPA^10^ - H-7 ^2^ - Y27632 ^11^ - Radicicol ^12^	Natural ligands include PECAM-1, β2 integrins, major class human rhinovirus. Others include affinity molecules binding to PECAM-1 or ICAM-1, etc.	From ≈50 nm to 5 μm
	Dymanin-independent (CLIC/GEEC, Arf6, flotillin-1)		Immune and endothelial cells, and various others depending on the marker	CD59 and other GPIAPs, MHCI	Recycling, endosomes and lysosomes	- Actin disruption - Filipin - Various depending on receptor	High dose of EGF?, anthrax toxin?, fibroblast growth factor 2 via syndecan 4, some GPIanchored proteins	Not fully characterized

^1^ Nocodazole interferes with the polymerization of microtubules; ^2^ Bisindolylmaleimide (BIM)-1 and H-7 inhibit PKC; ^3^ Wortmannin inhibits PI3 kinase; ^4^ Amiloride inhibits sodium–proton exchangers (NHEs), and other ion pumps; ^5^ Staurosporine is a broad-spectrum kinase inhibitor; ^6^ Monodansyl cadaverin (MDC) inhibits clathrin lattices arrangement; ^7^ Amantidine inhibits budding of clathrin-coated pits; ^8^ Cyclodextrin and filipin deplete and sequester, respectively, cholesterol in lipid rafts and caveolae; ^9^ Genistein inhibits tyrosine kinases; ^10^ Ethyl-isopropyl amiloride (EIPA) more specifically inhibits NHE1 in the plasmalemma; ^11^ Y27632 inhibits Rho-dependent kinase ROCK; ^12^ Radicicol inhibits Src kinase.

**Table 2:** Examples of clinical pharmaceutical agents effecting vesicular transport.

Pharmaceutical Agent		Application	Primary Molecular Targets	Observed Vesicular Trafficking Effects
**Agents that alter the cytoskeleton**	Vinca alkaloids (vinblastine, vincristine, vindesine, etc.)	Antineoplasic, e.g., non-small lung cancer, melanoma, testicular cancer, Hodgkin’s lymphoma, etc.	- At low concentrations they stabilize microtubule dynamics - At high concentration they lead to microtubule detachment from the organizing center, fragmentation, etc.	- Decreased uptake - Altered lysosomal transport - Enhanced fusion between autophagosomes and lysosomes in liver cells - Increased number of lysosomes in bile canaliculi cells, and decreased enzyme secretion
	Nocodazole			
	Colcemid			
	Colchicine	- Cancer - Gout - Familial Mediterranean fever - Behçetʹs disease		
**Lysosomotropic agents**	Choroquine	- Antimalaria agent. - Antiretroviral agent against HIV/AIDS - Chemosensitizing - Anti-inflammatory - Diabetes	Mild base avoiding lysosomal acidification and activation of lysosomal enzymes	- Alterations in vesicular formation and trafficking - Altered lipids in Rab5-positive endosomes - Endosomal enlargement - Altered trafficking and fusion of GLUT4 with the membrane
	Amiodarone	Arrythmia, such as in ventricular tachycardia or fibrillation, and other conditions	Inhibits voltage- and ligand-dependent potassium channel current	Engorgement and storage-like disease in several organs including the lungs, brain, skin, etc.
	Suramin	- African sleeping sickness - River blindness	Disruption of receptor–G protein coupling	Induction of lysosomal storage and alteration of associated vesicular transport
**Aminoglycosides antibiotics**	Streptomycin	Bacterial and mycobacterial infections	Unclear targets in eukaryotic cells, possibly mitochondrial ribosomes	Altered function of kidney lysosomes
	Kanamycin			
	Gentamicyn			
	Trospectomycin sulfate			- Trafficking defects - Formation of intracellular “lamellar bodies” in various organs
	Azithromycin			
**Lipophilic vitamins**	Vitamin E	- Antioxidant - Inflammation - Lysosomal storage disorders	- Modulates the plasmalemma curvature - Disrupts plasmalemma recruitment of PKC	- Enhanced exocytosis of lysosomes and their contents - Altered endocytic uptake

This table is not comprehensive, yet it provides relevant examples of different types of clinical phamaceuticals affecting vesicular transport.
